# A rapid and scalable density gradient purification method for *Plasmodium* sporozoites

**DOI:** 10.1186/1475-2875-11-421

**Published:** 2012-12-17

**Authors:** Mark Kennedy, Matthew E Fishbaugher, Ashley M Vaughan, Rapatbhorn Patrapuvich, Rachasak Boonhok, Narathatai Yimamnuaychok, Nastaran Rezakhani, Peter Metzger, Marisa Ponpuak, Jetsumon Sattabongkot, Stefan H Kappe, Jen CC Hume, Scott E Lindner

**Affiliations:** 1 Center for Mosquito Production and Malaria Infection Research, Seattle Biomedical Research Institute, 307 Westlake Avenue North, Suite 500, Seattle, WA, 98109, USA; 2 Malaria Program, Seattle Biomedical Research Institute, 307 Westlake Avenue North, Suite 500, Seattle, WA, 98109, USA; 3 Mahidol Vivax Research Center, Faculty of Tropical Medicine, Mahidol University, Bangkok, Thailand; 4 Department of Microbiology, Faculty of Science, Mahidol University, Bangkok, Thailand; 5 Department of Global Health, University of Washington, Seattle, WA, 98195, USA

**Keywords:** Sporozoite, Purification, Plasmodium, Falciparum, Vivax, Yoelii, Humanized mouse model

## Abstract

**Background:**

Malaria remains a major human health problem, with no licensed vaccine currently available. Malaria infections initiate when infectious *Plasmodium* sporozoites are transmitted by Anopheline mosquitoes during their blood meal. Investigations of the malaria sporozoite are, therefore, of clear medical importance. However, sporozoites can only be produced in and isolated from mosquitoes, and their isolation results in large amounts of accompanying mosquito debris and contaminating microbes.

**Methods:**

Here is described a discontinuous density gradient purification method for *Plasmodium* sporozoites that maintains parasite infectivity *in vitro* and *in vivo* and greatly reduces mosquito and microbial contaminants.

**Results:**

This method provides clear advantages over previous approaches: it is rapid, requires no serum components, and can be scaled to purify >10^7^ sporozoites with minimal operator involvement. Moreover, it can be effectively applied to both human (*Plasmodium falciparum*, *Plasmodium vivax*) and rodent (*Plasmodium yoelii*) infective species with excellent recovery rates.

**Conclusions:**

This novel method effectively purifies viable malaria sporozoites by greatly reducing contaminating mosquito debris and microbial burdens associated with parasite isolation. Large-scale preparations of purified sporozoites will allow for enhanced *in vitro* infections, proteomics, and biochemical characterizations. In conjunction with aseptic mosquito rearing techniques, this purification technique will also support production of live attenuated sporozoites for vaccination.

## Background

*Plasmodium* species, the aetiological agents of malaria, cycle between a mosquito vector and vertebrate host to complete its life cycle. Mammals become infected with malaria parasites when bitten by an infected *Anopheles* mosquito, which deposits the sporozoite form of the parasite into the host's skin (reviewed in [1]). Sporozoites then actively seek the host vasculature and upon invading it, passively migrate to the liver where they ultimately infect a hepatocyte and replicate within it. This liver stage of development (which lasts two days for rodent-infective species or six to eight days for human-infective species) results in the release of tens of thousands of red blood cell-infectious exoerythrocytic merozoites. These merozoites then initiate the blood stage of malaria infection that is associated with all clinical symptoms of the disease.

The characterization of sporozoite infectivity has often been limited by the presence of bacteria and yeast. Because of these contaminating microbes, the duration of many *in vitro* infections of permissive hepatocytes is often limited to only a few days. In addition, immunocompromised mice with humanized livers have recently been developed to provide an *in vivo* model of liver stage infections for both *Plasmodium falciparum* and *Plasmodium vivax* ( [2,3], Mikolajczak *et al.*, *personal communication*). Infections of these mice with large numbers of unpurified sporozoites can also systemically introduce large amounts of these microbes, which may negatively impact the health of these mice and affect the innate immune response against the parasite during the 6–8 day liver stage infection. These contaminants can similarly confound investigations of basic immunological responses of non-humanized mice against rodent malaria, as well.

Biochemical studies of sporozoites have also been hampered by relatively large amounts of residual mosquito debris compared to the parasite mass. For instance, proteomic studies of various *Plasmodium* species have used unpurified sporozoites and this has resulted in the identification of only a limited number of parasite-specific peptide hits, presumably due to the large number of contaminating mosquito-derived peptides [4-6]. Substantial reduction of mosquito proteins would substantially help such characterizations.

A previously published method of sporozoite purification requires that the parasites be first coated with bovine serum albumin (BSA) and then passed through ion exchange resin [7]. While this described procedure produces well-purified sporozoites, the fraction of sporozoites recovered is routinely low (30-40%) even with sporozoites obtained from isolated salivary glands (Lindner S., *unpublished results*). Moreover, this approach yields parasites coated with BSA, which is known to induce sporozoite apical organelle secretion and motility [8,9]. Another sporozoite purification method that employs BSA utilizes a discontinuous density gradient composed of diatrozoate sodium/Hypaque and several lengthy, high speed spins to purify sporozoites [10]. When injected into mice, these sporozoite preparations resulted in the death of significant numbers of the mice due to an immune response to BSA [11]. A revised protocol from the same authors that employed mouse serum in lieu of BSA did curtail mouse death, but did not reduce contaminating microbial burdens (ibid).

Ideally, an effective malaria vaccine will confer sterile protection prior to the onset of symptoms. The most promising subunit vaccine candidate, RTS,S, is currently in Phase 3 trials by GlaxoSmithKline but has shown only partial protection from subsequent infections [12]. Recent efforts have also focused on developing malaria vaccines comprised of live sporozoites attenuated by irradiation or genetic disruption, including the first trial testing syringe delivery of irradiated sporozoites [13-16]. These attenuated parasites are able to productively infect hepatocytes, but arrest at specific points within the liver stage of parasite development [17]. Interestingly, genetically attenuated parasites that arrest as late liver stage parasites (e.g. *pyfab b/f*^*-*^ parasites) provide greater and broader CD8+ T-cell responses and higher levels of protection than do radiation attenuated parasites or early arresting genetically attenuated parasites (ibid). Further studies and large-scale implementation of these vaccine candidates would greatly benefit from the production of large numbers of sporozoites that have been purified away from mosquito-derived contaminants to facilitate their characterization and deployment.

To address all of the above problems, herein is described a discontinuous density gradient purification method that employs a dense layer composed of Accudenz dissolved in water. This strategy effectively removes mosquito debris and hemocoel lipids, does not require BSA or serum, greatly reduces bacterial contamination, and eliminates yeast contamination. Moreover, this approach is applicable across all species of *Plasmodium* tested, including rodent-infective *Plasmodium yoelii* and human-infective *P. falciparum* and *P. vivax* sporozoites. Importantly, this method is scalable, as >10^7^ sporozoites can be routinely purified in a single gradient with excellent recovery rates. Lastly, this method is validated by demonstrating that these purified sporozoites are fully infectious in standard *in vitro* and/or *in vivo* infectivity assays for all three species. Taken together, this method provides a means to rapidly purify *Plasmodium* sporozoites for both basic research and vaccine production applications.

## Methods

### Accudenz column purification method

A 17% w/v solution of Accudenz (Accurate Chemical #AN7050) dissolved in distilled deionized water (ddH_2_O, Mediatech #25-055-CM) was filter sterilized and stored at 4°C. A 3 ml Accudenz cushion was loaded in a 15 ml conical tube and the dissected sporozoite mixture (up to 1 ml) was gently layered on top of the cushion. The column was spun at 2,500 *g* at room temperature for 20 minutes (no brake) and the interface (±) was transferred to a new, clean microcentrifuge tube and spun at top speed in a microcentrifuge for three minutes. The supernatant was aspirated and the pelleted sporozoites were resuspended in RPMI 1640 or Schneider’s insect medium and counted in a haemocytometer. Three or more biological replicates of all tested species of *Plasmodium* sporozoites (*Plasmodium yoelii*, *P. falciparum*, *P. vivax*) were tested to confirm sporozoite recovery.

### *Plasmodium yoelii* sporozoite production

Six-to-eight week old female Swiss Webster (“SW”) mice from Harlan (Indianapolis, IN) were used for production of wild-type *P. yoelii* 17XNL (“Py17XNL”, a non-lethal strain) blood stage parasites. Animal handling was conducted according to Institutional Animal Care and Use Committee approved protocols at the Seattle Biomedical Research Institute. Py17XNL parasites were cycled between SW mice and *Anopheles stephensi* mosquitoes that had been reared by standard methods (as described in Methods in Anopheles Research available at http://www.mr4.org). Infected mosquitoes were maintained on an 8% w/v dextrose solution in 0.05% w/v PABA water at 24°C and 70% humidity in 12 hour light/dark cycles. Mosquitoes were collected into sterile 70% v/v ethanol in ddH_2_O, rinsed in sterile ddH2O and finally washed in sterile 1xPBS. Salivary glands were individually isolated by microdissection in sterile RPMI media 14–16 days post-blood meal from batches of 50–75 mosquitoes, and were placed in a microfuge tube containing sterile RPMI media. Sporozoites were then released by three rounds of manual grinding of salivary glands (50 grinds per round), with glands re-pelleted at 100 *g* for 1 minute between rounds. The sporozoite-containing mixture was applied to the gradient as described above.

### *Plasmodium falciparum* sporozoite production

*In vitro P. falciparum* (NF54 strain) cultures were maintained in O+ erythrocytes grown in RPMI 1640 (25 mM HEPES, 2 mM L-glutamine) supplemented with 50 μm hypoxanthine and 10% A+ human serum in an atmosphere of 5% CO_2_, 5% O_2_, and 90% N_2_. Gametocyte cultures were initiated at 5% haematocrit and 0.8-1% parasitaemia (mixed stages) and maintained for up to 17 days with daily media changes.

Non-bloodfed adult female *An. stephensi* mosquitoes three to seven days post-emergence were collected into mesh-topped, wax-lined pots each containing up to 300 mosquitoes. Gametocyte cultures were quickly spun down and the pelleted infected erythrocytes were diluted to 40% haematocrit with fresh A+ human serum and O+ erythrocytes. Mosquitoes were allowed to feed upon these cultures through Parafilm for up to 20 minutes. Following blood feeding, mosquitoes were maintained for up to 19 days at 27°C, 75% humidity and were provided with an 8% w/v dextrose solution in 0.05% w/v PABA water. Salivary gland sporozoites were isolated as described above by microdissection 14–19 days post-blood meal.

### *Plasmodium vivax* sporozoite production

Blood samples from *P. vivax*-infected patients were collected at the Malaria Clinics (Kanchanaburi province, Thailand) in accordance with an approved human research protocol (protocol# TMEC 11–033, Faculty of Tropical Medicine, Mahidol University). Infected blood was heparinized and fed to five to seven-day old female *Anopheles dirus* mosquitoes using a membrane-feeding device [18]. Infected mosquitoes were maintained at 26°C, 75% humidity and were provided with a 10% w/v dextrose solution in water. Salivary gland sporozoites were isolated by microdissection as described above 15–18 days post-blood meal, but in batches of five mosquitoes at a time.

### Measurement of the reduction of bacterial contaminants

*Plasmodium yoelii* salivary gland sporozoites were dissected from three independent mosquito cages and were resuspended at 2×10^5^ sporozoites/1ml RPMI 1640 media. Sporozoites were either left untreated, or were incubated with 200 IU penicillin / 200 mg/ml streptomycin (Cellgro, Cat#30-002-CI) for 20 minutes at room temperature. Sporozoites from both conditions were then either left unpurified, or purified as described above. Sporozoites were pelleted, washed in RPMI 1640 to remove residual antibiotics, counted on a haemocytometer, serially diluted to 25000, 2500, 250, and 25 sporozoites in 100 μl RPMI 1640 and were plated on LB-agar plates. Plates were incubated at 37°C for 12–14 hours and colony-forming units per sporozoite plated were determined by manual counting.

### Assessment of *in vivo P. yoelii* sporozoite infectivity

*Plasmodium yoelii* salivary gland sporozoites were isolated from mosquito salivary glands, and 10^3^ or 10^4^ sporozoites were injected intravenously (“iv”) into the tail vein of SW mice. The time to blood stage patency (defined as >1 infected RBCs / 20,000 RBCs) was determined microscopically by Giemsa-stained thin blood smears. A one-day delay in the time to blood stage patency correlates with a 90% reduction in sporozoite infectivity [19]. Two biological replicates were measured for each condition.

### Assessment of *in vitro P. vivax* and *P. falciparum* sporozoite infectivity

HC-04, a human hepatocyte cell line, was used to produce liver stage infections [20]. Cells (2.5×10^4^) were seeded in each well of a 8-well chamber slides (LabTek, Campbell, CA) and incubated at 37°C with a complete medium (*P. vivax*: equal volumes of MEM and Ham’s F12 (Gibco BRL); *P. falciparum*: DMEM) supplemented with 10% foetal bovine serum, 100 U/ml penicillin, and 100 μg/ml streptomycin. At 24 h, the medium was aspirated and replaced with 2×10^4^ purified sporozoites in 250 μl of invasion medium (*P. vivax*: MEM:Ham’s F12 supplemented with 10% human AB serum, 100 U/ml penicillin, 100 μg/ml streptomycin, and 0.25 μg/ml fungizone; *P. falciparum*: complete media as above). After a four hour incubation, the invasion medium was aspirated and 300 μl of fresh complete medium (as above) was added to the infected hepatocytes. The culture was maintained for up to four days with daily media changes prior to visualization by an IFA (see below).

### Assessment of *in vivo P. falciparum* sporozoite infectivity

Female FRG NOD huHep mice with human chimeric livers (Yecuris Corp.) were infected with purified *P. falciparum* sporozoites by intravenous tail vein injection as previously described [3]. Six and seven days post-infection, mice were injected intravenously with 400 μl of packed O+ human RBCs. Mice were sacrificed two hours after the second RBC injection, and the blood was collected by cardiac puncture and placed into *in vitro* culture under standard conditions. Additionally, livers were simultaneously harvested and processed for IFA (see below).

### Indirect immunofluorescence assay (IFA)

Salivary gland sporozoites, *in vitro* and *in vivo* infected hepatocytes were processed for IFA essentially as described [21]. All sample types were stained at room temperature with phalloidin or the following appropriately diluted primary antibodies (rabbit anti-human fumaryl acetoacetate hydrolase (FAH, Yecuris), mouse anti-PfMSP1 (MR4), mouse anti-PfCSP (Clone 2A10), mouse anti-PvCSP210, mouse anti-PvCSP247, mouse anti-PbHsp70 (Clone 4C9) [22], Alexa Fluor- or FITC-conjugated secondary antibodies specific to mouse or rabbit IgG (Alexa Fluor 488, 594, Invitrogen; Dako), and 4^′^,6-diamidino-2-phenylindole (DAPI). Samples were covered with VectaShield (Vector Laboratories) and sealed under a coverglass slip. Fluorescent and DIC images were acquired using a DeltaVision Spectris RT microscope (Applied Precision) using a 40× or 100× oil objective and were deconvolved using the softWoRx software package, except for *P. vivax* liver stage parasites, which were acquired using a Nikon ECLIPSE 80i microscope with NIS-Elements F 3.0 software.

### Flow cytometric quantification of sporozoite invasion

Infection of HC-04 hepatocytes by *P. falciparum* sporozoites was measured as described previously [23]. Briefly, 4×10^5^ HC-04 cells were plated in each well of a 24-well plate one day prior to mock infection with dissection media or infection with 10^5^*P. falciparum* NF54 strain purified or unpurified sporozoites. Infection was allowed to proceed for 90 minutes, at which point hepatocytes were trypsinized, extensively washed, fixed, and permeabilized. Hepatocytes were blocked and then stained with an Alexa-Fluor 488-conjugated anti-PfCSP (2A10) to detect CSP-positive hepatocytes with a LSRII flow cytometer (Becton Dickinson) and FlowJo Software. Samples were normalized to the percent of hepatocytes infected by unpurified sporozoites (set at 100%).

### Statistics

A measurement of the statistical significance of sporozoite infectivity *in vitro* was conducted by a standard student’s t-test.

## Results

### Determination of critical purification parameters

**Figure 1:**
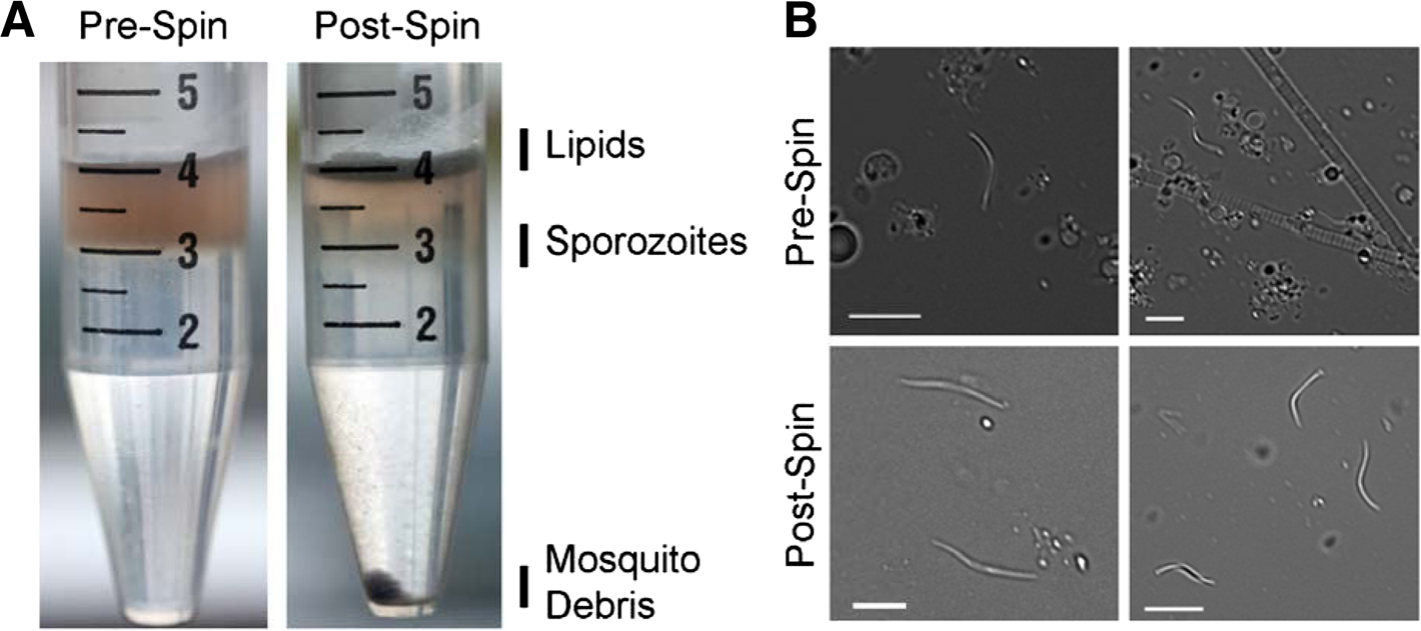
**Accudenz density gradient purification of *Plasmodium *sporozoites. A**. Photographs of a bilayer discontinuous Accudenz density gradient overlayed with *P. yoelii* sporozoites in RPMI before and after centrifugation. Mosquito debris pellets to the bottom of the gradient, mosquito lipids float to the top of the gradient, and sporozoites accumulate at the bilayer interface. **B**. Representative DIC images of unpurified and purified sporozoites before and after centrifugation show a significant reduction in mosquito material. Scale bar is 5 microns.

After sporozoite isolation from mosquito midguts or salivary glands, many sporozoite investigations are then hampered by excessive amounts of mosquito debris, microbes such as bacteria and yeast, or both. It would, therefore, be beneficial to devise a purification method to simultaneously solve both problems that would require minimal operator involvement (<5 minutes), be simple (one step), be rapid (20 minutes) and be scalable (able to purify millions of sporozoites simultaneously). To this end, several commercially available chemicals commonly used to create density gradients were assessed for their ability to purify sporozoites, and Accudenz-based gradients were identified as the top performer. Gradients that used Accudenz as the dense layer consistently produced the highest recovery of purified sporozoites from the bilayer interface (*P. yoelii*: 80.4% ± 11.6; *P. falciparum* 81.3% ± 5.74; *P. vivax* 61.9 ± 6.86), and have been used to successfully purify as few as 10^5^ sporozoites to over 2×10^7^ sporozoites in a single gradient with comparable sporozoite recoveries (Additional file 1). The Accudenz gradient also performed well in removing both mosquito debris (which forms a pellet at the base of the tube) and lipids (which float to the top of the gradient) (Figure 1A). Microscopically, the purity of these sporozoite samples was excellent as well, with little to no contaminating material visibly remaining after centrifugation (Figure 1B).

During the optimization of this purification method, three major parameters were identified that affected sporozoite recovery: the solvent used to produce the Accudenz cushion, the final concentration of Accudenz in the dense layer, and the relative centrifugal force applied to the gradient. *Plasmodium falciparum* sporozoites were loaded onto cushions of Accudenz dissolved in either 2× phosphate buffer saline (PBS), 1×PBS, 0.5×PBS or distilled, deionized water (ddH_2_O) and spun at 2,500 *g* for 20 minutes at room temperature (RT). A strong, dose-dependent negative effect on the percent recovery of sporozoites was observed with increasing concentrations of solutes present in PBS. In contrast, using ddH_2_O as the solvent allowed for consistently high percentage recoveries of sporozoites, and was used for all subsequent experiments (Additional file 2). The optimal concentration of Accudenz for use in the dense layer (17%) was determined by testing a range of concentrations (5, 10, 17, 25% w/v in ddH_2_O) with *P. falciparum* sporozoites (Additional file 3).

Additionally, using both sporozoite recovery and amounts of residual mosquito debris as metrics, various relative centrifugal forces (1,500 *g*, 2,000 *g*, 2,500 *g*, 3,000 *g*) were tested upon the gradient and it was determined that optimal recovery and purification was obtained at 2,500 *g* (Additional file 4). Thus, the optimal conditions for sporozoite purification employ a 17% w/v Accudenz in ddH_2_O cushion under the dissected sporozoite mixture (in either RPMI 1640 or Schneider’s Medium), spun at 2,500 *g* for 20 minutes at room temperature.

### Reduction/elimination of contaminating microbial burdens

Using these optimized purification conditions, it was determined if this method would significantly reduce the amounts of contaminating bacteria and yeast remaining with the purified sporozoites. To this end, *P. yoelii* salivary gland sporozoites were either left unpurified or were purified over an Accudenz cushion as described. Additionally, to test if pre-treatment of the sporozoites with antibiotics would further reduce the microbial burden, samples were incubated with penicillin/streptomycin or left untreated. Serial dilutions of sporozoites from each of these four conditions were plated on LB agar plates, the number of colony forming units (CFUs) per sporozoite was counted (Table 1). No significant reduction in CFUs was achieved by pre-treatment of sporozoites with antibiotics alone. In contrast, Accudenz purification greatly reduced bacterial contamination (97.5% + 0.757) associated with sporozoites, which was further affected by treatment with penicillin and streptomycin (99.0% + 0.252). In confirmation of this, an equal number of unpurified or purified *P. yoelii* (10^5^), *P. falciparum* (10^5^), or *P. vivax* (2×10^4^) sporozoites were inoculated into complete DMEM or RPMI 1640 media in independent wells of 6-well tissue culture plates at 37°C with 5% CO_2_. All wells containing unpurified sporozoites became overgrown (visible by eye and/or microscopically) with bacteria (within 1–2 days) or yeast (within 4 days, data not shown), whereas wells containing purified sporozoites routinely remained free from contamination for at least 1 week.

**Table 1:** **Measurements of the reduction of bacterial contaminants by penicillin/streptomycin pre-treatment and Accudenz of *P. yoelii *sporozoites**

	-	-	+	+	**Accudenz Purification**
	-	+	-	+	**Penicillin**/**Streptomycin**
Replicate 1	0.84	0.68	0.017 (98.0%)	0.0080 (99.0%)	
Replicate 2	1.2	2.1	0.040 (96.6%)	0.0080 (99.3%)	
Replicate 3	1.0	1.6	0.022 (97.8%)	0.012 (98.8%)	

Salivary gland sporozoites were resuspended in RPMI 1640, left untreated or treated with penicillin/streptomycin, and were left unpurified or were purified on an Accudenz discontinuous gradient. Sporozoites were pelleted and washed in RPMI 1640, and then serially diluted and plated on LB-agar plates. Total colony forming units per sporozoite are listed for three biological replicates. Percent reductions of residual bacterial burdens for purified sporozoites compared to unpurified sporozoites not treated with penicillin/streptomycin are listed in parenthesis.

### Purified *P. yoelii* sporozoites are fully infectious to mice

Having demonstrated that this method readily produces highly purified *Plasmodium* sporozoites, it was critical to next determine if these sporozoites remained infectious. This was first assessed by testing for any significant loss of infectivity with the rodent-infective *P. yoelii* malaria parasite, as this can be easily measured by the presence of a delay in the appearance of blood stage parasites (termed a “delay in blood stage patency”) after the intravenous (iv) injection of salivary gland sporozoites. Using naïve outbred Swiss Webster (SW) mice, 10^3^ or 10^4^ unpurified or purified sporozoites were iv injected and the time to blood stage patency (defined as >1 Giemsa-positive, infected RBC per 20,000 RBCs on a thin blood smear) was measured. All mice infected with either purified or unpurified sporozoites developed blood stage patency at the same time point after infection (depending on the initial dose), indicating that no detectable loss of sporozoite infectivity (e.g. less than 10-fold) was produced by this purification method nor by sporozoite pre-treatment with penicillin/streptomycin (Table 2).

**Table 2:** **Measurements of the time to blood stage patency indicate that there is no loss of infectivity with purified *P. yoelii *sporozoites**

**Type**	**Replicate** #	# **Sporozoites injected**	# **Mice infected**	# **Mice patent** (**Day of patency**)
Unpurified	1	10,000	5	5 (3d)
	2	10,000	5	5 (3d)
	1	1,000	5	5 (4d)
	2	1,000	5	5 (4d)
Purified	1	10,000	5	5 (3d)
	2	10,000	5	5 (3d)
	1	1,000	5	5 (4d)
	2	1,000	5	5 (4d)

Salivary gland sporozoites were isolated from mosquitoes at 14d post-blood meal and injected intravenously into groups of five naïve Swiss Webster mice in duplicate. Time to blood stage patency (defined as >1 parasite per 20,000 RBCs) was determined microscopically by daily Giemsa-stained thin blood smears, and was identical for sporozoites pretreated with antibiotics or for those left untreated.

### Purified *P. falciparum* sporozoites have no loss of infectivity *in vitro*

**Figure 2:**
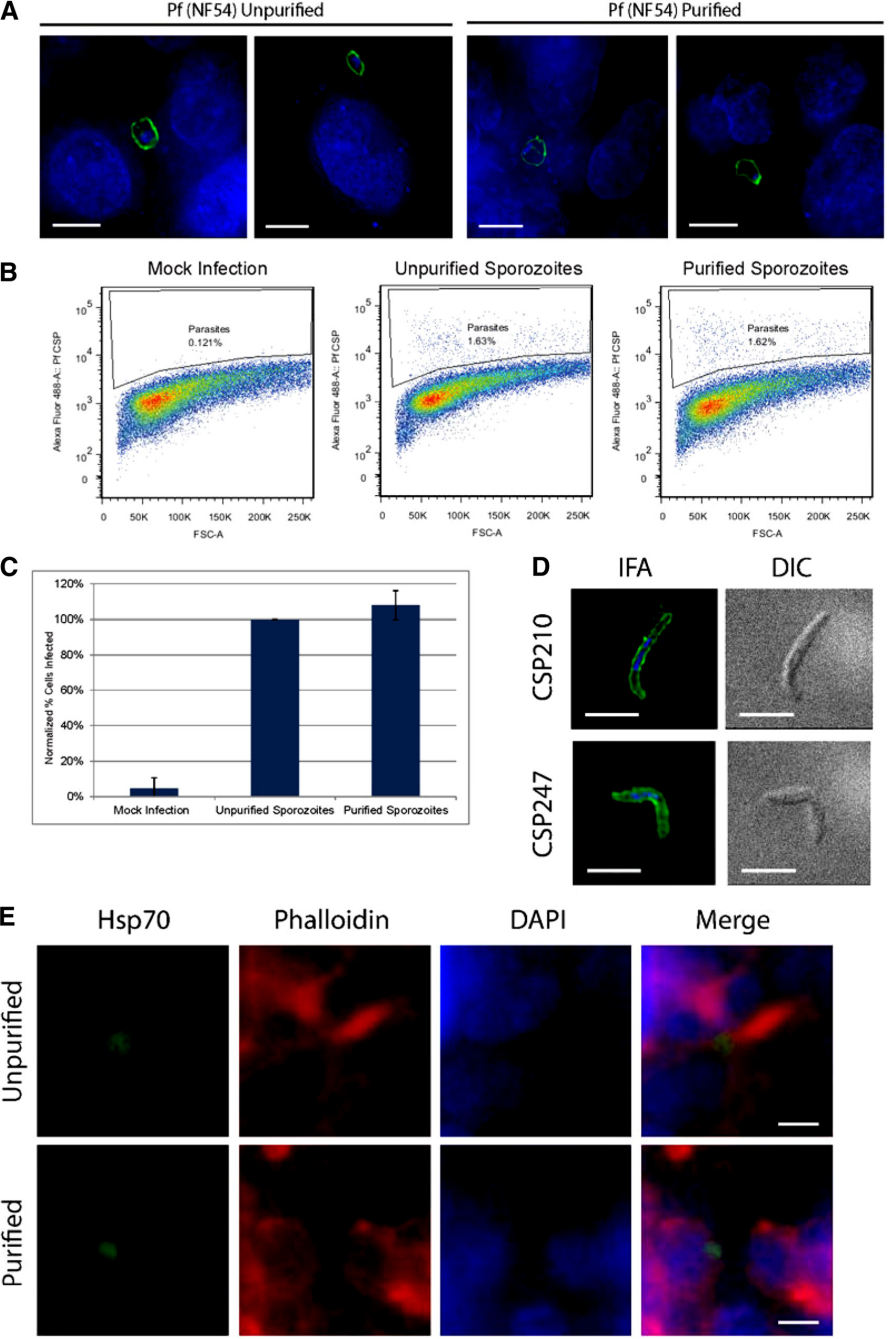
**Purified *P. falciparum *and *P. vivax *sporozoites remain infectious *in vitro*. A**. HC-04 cells were infected with unpurified or purified P. falciparum sporozoites for 24 hours and were then subjected to an IFA (green: anti-PfCSP (2A10), blue: DAPI). Scale bar is 10 microns. **B**. Representative plots of sporozoite infectivity are provided from flow cytometric measurements of anti-Pf CSP antibody staining of HC-04 cells **C**. A graphical representation of two biological replicates of infections from (B) demonstrates that there is no infectivity defect of purified sporozoites. **D**. Purified *P. vivax* sporozoites were observed by IFA (green: anti-PvCSP 210 or anti-PvCSP247, blue: DAPI). No gross differences in parasite morphology were detected. Scale bar is 5 microns. **E**. HC-04 cells were infected with unpurified and purified *P. vivax* sporozoites for four days and developing liver stage parasites were observed by IFA (green: anti-PbHsp70, red: Phalloidin, blue: DAPI). No gross differences in parasite morphology were detected. Scale bar is 5 microns.

As purification of sporozoites over an Accudenz gradient significantly reduced the contaminating microbial burden found associated with sporozoites, it would also be important to determine if there was any effect upon the infectivity of *P. falciparum* sporozoites for hepatocytes *in vitro*. The human hepatocarcinoma cell line HC-04 was mock infected without sporozoites, or incubated with unpurified or purified sporozoites. Observations of infected hepatocytes by an indirect immunofluorescence assay (IFA) one day post-infection demonstrated no significant difference in parasite morphology with purified or unpurified sporozoites (Figure 2A). Moreover, while wells incubated with unpurified sporozoites readily became contaminated within two to three days, wells that received purified sporozoites remained contamination-free for the complete length of the assay (typically at least one week). Additionally, the fraction of infected hepatocytes was measured shortly after sporozoite infection (90 minutes) by using a recently published flow cytometric assay [23]. This assay allows precise quantifications of infection by analysing large numbers of cells (10^5^) for the presence of the most abundant sporozoite protein, circumsporozoite protein (CSP).

Representative plots (Figure 2B) and a normalized graph of duplicate biological replicates (Figure 2C) of mock infected cells, or cells infected with unpurified or purified sporozoites indicate that no significant difference (measured by a student’s t-test) in *P. falciparum* sporozoite infectivity exists between purified and unpurified parasites *in vitro*.

### Purified *P. vivax* sporozoites retain infectivity *in vitro*

This purification technique was also tested to determine if it could be extended to *P. vivax* sporozoites, another major human-infective malaria species. Salivary gland sporozoites (both CSP210 and CSP247 parasite types [24]) were dissected from infected *Anopheles dirus* mosquitoes by standard methods, and were purified using standard optimized conditions. Consistently 55-69% of the input sporozoites were recovered with a comparable elimination of mosquito debris as with other species. Both the CSP210 and CSP247 types of purified *P. vivax* sporozoites were examined by IFA and found to retain normal morphology and antibody staining patterns (Figure 2D). The ability of purified and unpurified sporozoites to productively infect HC-04 hepatocytes *in vitro* was also tested. Mixed populations of CSP210 and CSP247 types of *P. vivax* sporozoites were allowed to infect and develop for four days, and were then observed by IFA. In both conditions, a comparable number of appropriately developing liver stage parasites were detected (94.2% ± 16.6, Figure 2E). This indicates that as with *P. yoelii* and *P. falciparum*, purified *P. vivax* sporozoites retain their infectivity for hepatocytes. Moreover, it was also observed that Accudenz purification routinely removed all traces of yeast contamination from the sporozoites, whereas unpurified sporozoites lead to the well becoming overgrown with yeast within 4 days. This sporozoite purification method should now enable long-term *in vitro* cultures, especially for the study of hypnozoites, to be routinely maintained contamination-free.

### Purified *P. falciparum* sporozoites productively complete liver stage development in a humanized mouse model and transition to a sustainable blood stage infection

**Figure 3:**
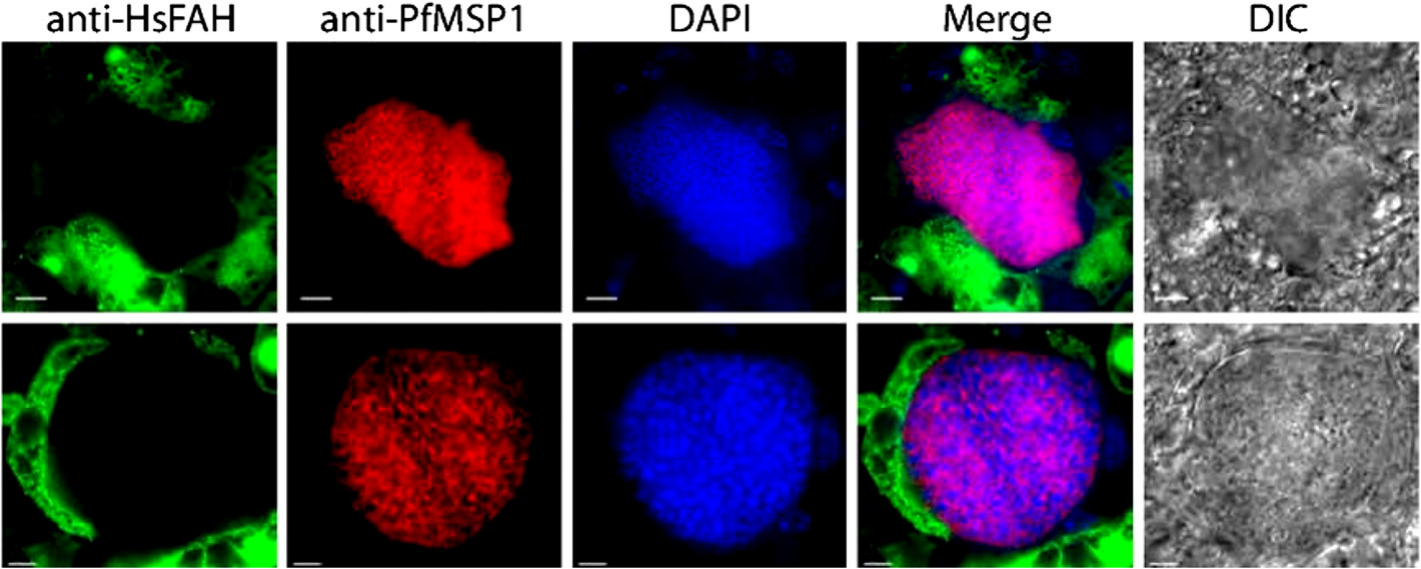
**Purified *P. falciparum *sporozoites remain infectious to a humanized mouse.** Two representative IFA panels of late liver stage *P. falciparum* parasites are shown using antibodies to human FAH (green), PfMSP1 (red) or with DAPI (blue). These images indicate that purified sporozoites are able to fully progress through liver stage development. Scale bar is 10 microns.

It has recently been shown that the FRG NOD huHep mouse, a human-liver chimeric mouse, can support complete *P. falciparum* liver stage development [3]. Additionally, these mice were competent to allow a liver stage to blood stage transition [3]. Since the FRG NOD huHep mouse is immunocompromised, it could be surmised that injection of these mice with purified sporozoites would reduce the bacterial and fungal burden on the mice, which could affect the health of the mice and possibly liver stage development. Therefore, two FRG NOD huHep mice were injected intravenously with 3 × 10^6^ purified *P. falciparum* sporozoites. Mice were injected with human red blood cells as previously described and the mice were euthanized seven days after sporozoite infection [3]. The blood removed from the mice was transitioned into a *P. falciparum* asexual culture and indeed, a healthy *in vitro* asexual culture of transitioned *P. falciparum* blood stages was achieved. The livers were also removed from the euthanized mice, fixed and subjected to an IFA as previously described [25]. Using antibodies to parasite merozoite surface protein 1 (MSP1) and human fumaryl acetoacetate hydrolase (FAH) (present only in the human hepatocytes within the FRG NOD huHep mouse liver), as expected, mature liver stages were observed to be present in the livers of the inoculated mice (Figure 3). Thus, the purified *P. falciparum* sporozoites appear to behave no differently to the unpurified *P. falciparum* sporozoite in this *in vivo* assay of liver stage development and *ex vivo*/*in vitro* assay of liver stage to blood stage transition.

## Discussion

This work describes and demonstrates that an Accudenz density gradient purification method of *Plasmodium* sporozoites produces parasites that remain viable and infective. A discontinuous gradient approach was favored over using a continuous gradient, as the former also acts to localize and concentrate sporozoites at the interface of the light and dense layers. This approach thus removes the requirement to collect and assess multiple samples for the presence of sporozoites, which is needed when using continuous gradients as sporozoites distribute throughout a larger portion of the gradient [26,27]. Moreover, this rapid and technically simple method successfully removes mosquito debris and lipids, as well as contaminating bacterial and fungal contaminants. Importantly, this is accomplished in the absence of serum or any serum components, which are known to induce sporozoite secretion and can cause immune responses in mice. Lastly, this approach was successfully scaled up and allowed the recovery of >10^7^ sporozoites from a single density gradient. This purification method was validated for both rodent-infective and human-infective malaria species, as purified sporozoites perform identically to unpurified sporozoites by all *in vitro* and *in vivo* assays used to date.

Of special interest was the implementation of these purified sporozoites with humanized mouse models. Injection of purified sporozoites into the immunocompromised FRG NOD huHep mouse could be most beneficial due to the removal of contaminating bacteria and fungi which could cause inflammatory responses in the mice and even an uncontrollable infection leading to their death. Inflammation could cause the death of *P. falciparum* liver stage parasites early after infection. If comparative studies were to be made on liver stage infectivity in the FRG huHep mouse model, purification of sporozoites would be vital to exclude the differing levels of microbial burden between sporozoite samples.

Also, because this method completely eliminated yeast contamination of *P. vivax* sporozoites, long-term *in vitro* assays should no longer be impacted by this contaminant. This is of particular interest for investigations of liver stage development of *P. vivax*. Not only will this enable easier characterization of the actively growing forms of the liver stage parasite, which take approximately 8 days to fully develop *in vivo*, but perhaps also studies of the poorly characterized “dormant forms” (termed hypnozoites) of the parasite. Hypnozoites are a major reservoir of viable parasites in infected individuals, which can persist for years and likely substantially hinder efforts to eradicate the disease.

In addition to the infectivity assays described here, it is anticipated that this purification method will greatly facilitate biochemical and molecular biological investigations that were hindered by the high amounts of microbial contamination and/or mosquito debris. For instance, previously reported proteomic efforts of sporozoites have been limited by contaminating mosquito proteins, which required large numbers of mass spectrometric runs to identify hundreds of parasite proteins. In contrast, recent proteomic efforts with purified sporozoites have overcome this limitation, producing the most comprehensive proteomes of this stage (~1,900-2,000 proteins), with >86% of proteins identified by multiple unique peptide hits (Lindner and Swearingen *et al.* submitted). Further investigations of sporozoite biology will no doubt benefit from these approaches as well.

Lastly, with the development of live attenuated sporozoites as potential malaria vaccines come several technical hurdles should a lead candidate be identified in clinical trials for large-scale implementation. One such hurdle is the effective removal of contaminating mosquito debris associated with the sporozoites. This density gradient purification method should meet several of the metrics required for this process (e.g. purity, infectivity, scalability), and may prove useful to streamline these efforts. When combined with aseptic mosquito rearing techniques, this method could make syringe administration of live attenuated sporozoite immunizations practical. All told, this novel, cross-species purification method holds great promise for accelerating both basic research of sporozoite biology and translational efforts to use the sporozoite medically.

## Conclusions

Described in this study is a discontinuous density gradient method that rapidly and effectively purifies malaria sporozoites by greatly reducing contaminating mosquito debris and microbial burdens associated with parasite isolation. Moreover, the viability of these sporozoites was confirmed, and the application of this approach to commonly used *in vitro* and *in vivo* assays was demonstrated. Large-scale preparations of sporozoites purified by this approach will allow for enhanced *in vitro* infections, proteomics, and biochemical characterizations. Finally, this purification method, in conjunction with aseptic mosquito rearing techniques, will support production of live attenuated sporozoites that will be suitable for vaccination.

## Abbreviations

BSA: Bovine Serum Albumin; IFA: Indirect Immunofluorescence Assay; FAH: Fumaryl Acetoacetate Hydrolase; CSP: Circumsporozoite Protein; MSP1: Merozoite Surface Protein 1; FITC: Fluorescein isothiocyanate; DAPI: 4^′^,6-Diamidino-2-Phenylindole; DIC: Differential Interference Contrast; PBS: Phosphate Buffer Saline; CFUs: Colony Forming Units; SW: Swiss Webster.

## Competing interests

A provisional patent has been filed on this purification method on August 1^st^, 2012 (US Patent Application Number 61678541).

## Authors’ contributions

Developed purification method: MK, SEL; Assessed *P. yoelii* purification and infectivity: MK, MF; Assessed *P. falciparum* purification and infectivity: AMV, NR, PM, SEL; Assessed *P. vivax* purification and infectivity: RP, RB NY and SEL; Assessed reductions of microbial burden: MF; Provided project supervision: MP, JS, SHK, JCCH, SEL; Wrote the Manuscript and Analysed the Data: MK, AMV, JCCH, SEL. All authors read and approved the final manuscript.

## Supplementary Material

Additional file 1 Representative pre- and post-purification sporozoite numbers tested in optimal conditions. Click here for file

Additional file 2 The composition of the solvent used to produce the Accudenz gradient greatly affects sporozoite recovery. Click here for file

Additional file 3 Representative pre- and post-purification sporozoite numbers tested with different accudenz concentrations. Click here for file

Additional file 4 The relative centrifugal force applied to the Accudenz gradient greatly affects sporozoite recovery. Click here for file
