# Supplementary material for: A rapid and scalable density gradient purification method for Plasmodium sporozoites
Source: Malar J. 2012 Dec 17;11:421. doi: 10.1186/1475-2875-11-421 (PMC3543293; doi:10.1186/1475-2875-11-421)
Supplement: Additional file 1 — Representative pre- and post-purification sporozoite numbers tested in optimal conditions. [file 1475-2875-11-421-S1.pdf]

Additional file 1: Representative Pre and Post-Purification Sporozoite Numbers Tested in Optimal Conditions

Plasmodium yoelii sporozoites

Trial #	# Sporozoites Pre-Purification	# Sporozoites Post-Purification	% Recovery
1	1.14E+07	1.03E+07	90.4
2	9.56E+06	6.76E+06	70.7
3	8.90E+06	8.10E+06	91.0
4	8.40E+06	7.10E+06	84.5
5	7.76E+06	6.70E+06	86.3
6	5.60E+06	5.21E+06	93.0
7	4.50E+06	2.90E+06	64.4
8	2.10E+06	1.40E+06	66.7
9	1.30E+06	8.75E+05	67.3
10	1.00E+05	8.95E+04	89.5

Average % Recovery	80.4 +/- 11.6

Plasmodium falciparum sporozoites

Trial #	# Sporozoites Pre-Purification	# Sporozoites Post-Purification	% Recovery
1	2.12E+07	1.67E+07	78.9
2	1.32E+07	9.87E+06	74.5
3	9.56E+06	7.86E+06	82.2
4	7.45E+06	6.81E+06	91.4
5	6.57E+06	5.37E+06	81.7
6	6.46E+06	5.20E+06	80.5
7	5.40E+06	4.30E+06	79.6
8	4.50E+06	4.08E+06	90.7
9	5.00E+05	3.74E+05	74.8
10	3.00E+05	2.35E+05	78.3

Average % Recovery	81.3 +/- 5.74

Plasmodium vivax sporozoites

Trial #	# Sporozoites Pre-Purification	# Sporozoites Post-Purification	% Recovery
1	2.50E+06	1.37E+06	54.8
2	2.22E+06	1.52E+06	68.5
3	1.33E+06	8.30E+05	62.4

Average % Recovery	61.9 +/- 6.86
