# Supplementary material for: A rapid and scalable density gradient purification method for Plasmodium sporozoites
Source: Malar J. 2012 Dec 17;11:421. doi: 10.1186/1475-2875-11-421 (PMC3543293; doi:10.1186/1475-2875-11-421)
Supplement: Additional file 2 — The composition of the solvent used to produce the Accudenz gradient greatly affects sporozoite recovery. [file 1475-2875-11-421-S2.pdf]

Additional file 2: The composition of the solvent used to produce the Accudenz gradient greatly affects sporozoite recovery.

Plasmodium falciparum sporozoites

**Solvent**	**% Recovery Trial 1**	**% Recovery Trial 2**	**Average**
Water	77.8	93.2	85.5
0.5x PBS	27.4	24.2	25.8
1.0x PBS	22.4	14.4	18.4
2.0X PBS	1.4	0.9	1.15
