# Supplementary material for: A rapid and scalable density gradient purification method for Plasmodium sporozoites
Source: Malar J. 2012 Dec 17;11:421. doi: 10.1186/1475-2875-11-421 (PMC3543293; doi:10.1186/1475-2875-11-421)
Supplement: Additional file 3 — Representative pre- and post-purification sporozoite numbers tested with different accudenz concentrations. [file 1475-2875-11-421-S3.pdf]

Additional file 3: Representative Pre and Post-Purification Sporozoite Numbers Tested with Different Accudenz Concentrations

Plasmodium falciparum sporozoites

% w/v Accudenz Concentration	# Sporozoites Pre-Purification	# Sporozoites Post-Purification	% Recovery
5	5.00E+05	7.40E+04	14.8
10	5.00E+05	2.18E+05	43.5
17	5.00E+05	4.17E+05	83.4
25	5.00E+05	4.43E+05	88.5
