# Supplementary material for: A rapid and scalable density gradient purification method for Plasmodium sporozoites
Source: Malar J. 2012 Dec 17;11:421. doi: 10.1186/1475-2875-11-421 (PMC3543293; doi:10.1186/1475-2875-11-421)
Supplement: Additional file 4 — The relative centrifugal force applied to the Accudenz gradient greatly affects sporozoite recovery. [file 1475-2875-11-421-S4.pdf]

Additional file 4: The relative centrifugal force applied to the Accudenz gradient greatly affects sporozoite recovery.

Plasmodium yoelii

Relative Centrifugal Force	% Recovery
1500 xg	53.4
2000 xg	62.8
2500 xg	91.7
3000 xg	12.0

Plasmodium falciparum

Relative Centrifugal Force	% Recovery
1500 xg	48.0
2000 xg	61.0
2500 xg	88.9
3000 xg	8.5
